# Negative regulation and developmental competence in *Aspergillus*

**DOI:** 10.1038/srep28874

**Published:** 2016-07-01

**Authors:** Mi-Kyung Lee, Nak-Jung Kwon, Im-Soon Lee, Seunho Jung, Sun-Chang Kim, Jae-Hyuk Yu

**Affiliations:** 1 Department of Bacteriology, The University of Wisconsin-Madison, Madison, Wisconsin 53706, USA; 2 Department of Biological Sciences and Center for Biotechnology Research, Institute for Ubiquitous Information Technology and Applications (UBITA), Konkuk University, Seoul 143-701, Republic of Korea; 3 Department of Bioscience and Biotechnology and Center for Biotechnology Research, Institute for Ubiquitous Information Technology and Applications (UBITA), Konkuk University, Seoul 143-701, Republic of Korea; 4 Department of Biological Sciences, Korea Advanced Institute of Science and Technology, Dae-Jeon, Republic of Korea

## Abstract

Asexual development (conidiation) in the filamentous fungus *Aspergillus nidulans* is governed by orchestrated gene expression. The three key negative regulators of conidiation SfgA, VosA, and NsdD act at different control point in the developmental genetic cascade. Here, we have revealed that NsdD is a key repressor affecting the quantity of asexual spores in *Aspergillus*. Moreover, nullifying both *nsdD* and *vosA* results in abundant formation of the development specific structure conidiophores even at 12 h of liquid culture, and near constitutive activation of conidiation, indicating that acquisition of developmental competence involves the removal of negative regulation exerted by both NsdD and VosA. NsdD’s role in repressing conidiation is conserved in other aspergilli, as deleting *nsdD* causes enhanced and precocious activation of conidiation in *Aspergillus fumigatus* or *Aspergillus flavus*. *In vivo* NsdD-DNA interaction analyses identify three NsdD binding regions in the promoter of the essential activator of conidiation *brlA*, indicating a direct repressive role of NsdD in conidiation. Importantly, loss of *flbC* or *flbD* encoding upstream activators of *brlA* in the absence of *nsdD* results in delayed activation of *brlA*, suggesting distinct positive roles of FlbC and FlbD in conidiation. A genetic model depicting regulation of conidiation in *A. nidulans* is presented.

Asexual development (conidiation) in the fungal class Ascomycetes results in the formation of mitotically derived conidiospores, or conidia^1^. Despite a great variety in conidial form and function, all conidia represent non-motile asexual propagules that are usually made from the side or tip of specialized sporogenous cells, i.e., phialides in *Aspergillus*, via asymmetric mitotic cell division^1^.

The genetic mechanisms of conidiation have been extensively studied in the model fungus *Aspergillus nidulans*. In a simple way, the *A. nidulans* asexual reproductive cycle can be divided into four distinct stages, beginning with a growth phase, proceeding through initiation of the developmental pathway, execution of the developmentally regulated events leading to sporogenesis, and concluding with switching off conidiation by feed-back control^2^. The growth phase involves germination of a conidium and formation of an undifferentiated network of interconnected hyphal cells that form the mycelium. After a certain period of vegetative growth, under appropriate conditions, some of the hyphal cells stop normal growth and begin conidiation by forming complex structures called conidiophores that bear multiple chains of conidia (Fig. 1A; reviewed in ref. 1).

Conidiation does not usually occur in *A. nidulans* until cells have gone through a defined period of vegetative growth necessary for cells to acquire the ability to respond to development signals, which is defined as a competence^3^. Under normal media conditions, *A. nidulans* can be maintained in the vegetative stage of its life cycle by growing hyphae submerged in liquid medium. In liquid submerged culture, conidiation hardly takes place and sexual fruiting never occurs unless vegetative cells are exposed to air. Previous studies have revealed that *A. nidulans* cells require approximately 18 h of growth before they are competent to respond to the inductive signal provided by exposure to air^3^,^4^.

A key event responding to the developmental inductive signal is activation of *brlA*, which encodes a C_2_H_2_ zinc finger transcription factor (TF) (Fig. 1B)^5^. Further genetic and biochemical studies have identified the *abaA* and *wetA* genes as necessary regulators of conidiation. The *abaA* gene encodes a putative TF that is activated by *brlA* during the middle stages of conidiophore development after differentiation of metulae^6^,^7^. The *wetA* gene, activated by AbaA, functions in late phase of conidiation for the synthesis of crucial cell wall components and conidial metabolic remodeling^8^,^9^. These three genes have been proposed to define a central regulatory pathway that acts in concert with other genes to control conidiation-specific gene expression and determine the sequence of gene activation during conidiophore development and spore maturation^10^,^11^,^12^ (reviewed in ref. 1).

Subsequent studies have identified various upstream developmental activators (UDAs), *fluG*, *flbA*, *flbB*, *flbC*, *flbD*, and *flbE* that influence *brlA* expression (Fig. 1B)^13^,^14^,^15^. Mutations in any of these genes result in “fluffy” colonies that are characterized by undifferentiated cotton-like masses of vegetative cells (reviewed in ref. 1). Each of the FlbB, FlbC and FlbD proteins contains a DNA binding domain and they are shown to be direct activators of *brlA* expression^16^,^17^. The two genetic cascades composed of *fluG* →→ *flbE*/*flbB*→ *flbD* → *brlA*, and *fluG* →→ *flbC* → *brlA* were proposed, in which *fluG* functions upstream^18^.

Our studies to further understand the developmental control mechanisms have identified three key negative regulators of conidiation, SfgA, VosA, and NsdD^19^,^20^,^21^. The *fluG* suppressor *sfgA* is predicted to encode a Zn(II)_2_Cys_6_ domain protein, and positioned between FluG and FLBs (Fig. 1B)^16^,^22^. The *velvet* domain TF VosA and the GATA-type TF NsdD were isolated via gain-of-function genetic screens as repressors of conidiation^19^,^21^. VosA, which is activated by AbaA, governs spore maturation and exerts negative feedback regulation of *brlA* by binding to the 11 nucleotide VosA responsive element (VRE) in the *brlAβ* promoter^19^,^23^. NsdD, initially identified as a key activator of sexual fruiting^24^, was found to be also a key repressor of conidiation^21^. The deletion of *nsdD* bypasses the needs for FluG and all UDAs, but not *brlA*, for conidiation, indicating that NsdD acts downstream of UDAs and upstream or at the same level of *brlA*^25^.

In the present study, we further investigate negative regulation of conidiation and developmental competence. Through combinatorial genetic studies, we have found that VosA and NsdD are the major factors repressing *brlA* expression, and thereby influencing the acquisition of developmental competence in *A. nidulans*. We also report that the repressive role of NsdD in conidiation is conserved in other aspergilli. In *A. nidulans* conidia, NsdD directly binds to the *brlAβ* promoter region, which contains a GATAA sequence potentially interacting with NsdD. We also demonstrate that FlbC and FlbD are necessary for full activation of *brlA* even in the absence of *nsdD*. A working genetic model depicting the positive and negative regulations of *brlA* expression and conidiation in *A. nidulans* is presented.

## Results

### NsdD is a key factor determining the number of conidia

**Figure 1:**
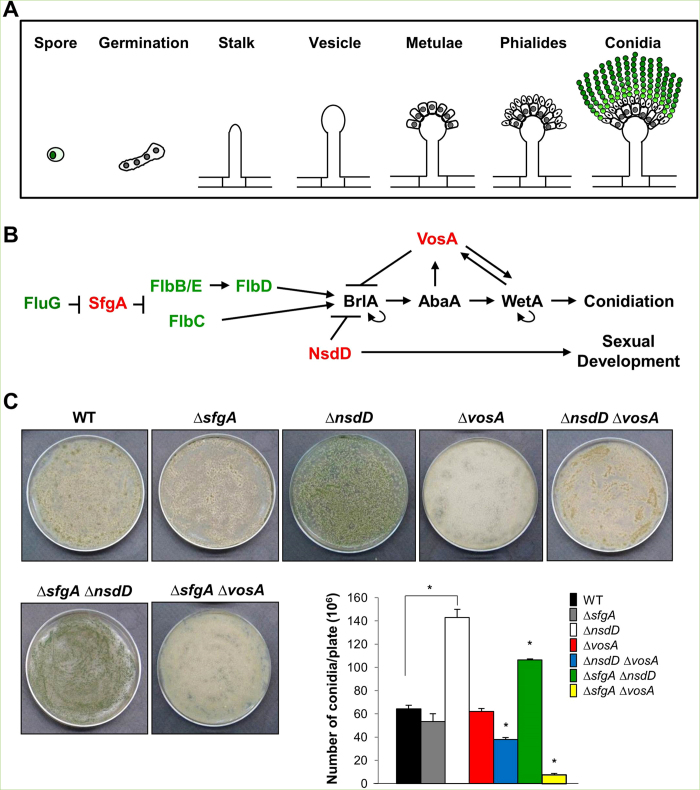
Background information and quantitative analyses of conidiation. (**A**) A schematic presentation of development of conidiophore in *A. nidulans*. (**B**) A genetic model for developmental regulation. Greens are activators and reds are repressors of *brlA*. (**C**) Conidiation levels in WT and various mutants. About 10^5^ conidia of WT (FGSC4), Δ*sfgA* (TNJ57), Δ*nsdD* (TNJ108), Δ*vosA* (THS15), Δ*nsdD* Δ*vosA* (TMK11), Δ*sfgA* Δ*nsdD* (TMK5), and Δ*sfgA* Δ*vos* (TMK10) strains were spread on solid MMG and grown for 2 days and the numbers of conidia per plate were counted in triplicates (*P < 0.001).

Previously, we showed that *vosA* and *nsdD* play an additive role in repressing conidiation and *brlA* expression in vegetative cells^21^. To further expand our understanding on the genetic interactions of the three negative regulators, we generated double mutants: ∆*nsdD* ∆*vosA*, ∆*sfgA* ∆*nsdD* and ∆*sfgA* ∆*vosA*. We then quantified the conidiation levels of FGSC4 (wild type; WT), ∆*sfgA*, ∆*nsdD*, ∆*vosA*, ∆*nsdD* ∆*vosA*, ∆*sfgA* ∆*nsdD* and ∆*sfgA* ∆*vosA* strains by spreading conidia onto solid MMG and incubating for 2 days. As shown in Fig. 1C, the ∆*nsdD* mutant produced ~2.3 fold more conidia than WT and other mutant strains (p < 0.001). The ∆*sfgA* ∆*nsdD* double mutant produced less number of conidia than the ∆*nsdD* single mutant, but more than WT (p < 0.001). On the contrary, the ∆*sfgA* ∆*vosA* mutant produced a highly reduced number of conidia, ~6 fold less than WT and the ∆*vosA* mutant. The ∆*nsdD* ∆*vosA* double mutant produced a similar number of conidia to WT. These results suggest that NsdD is a major determinant of the number of conidia being produced on solid culture condition. The ∆*sfgA* ∆*vosA* mutant exhibited a highly reduced number of conidia than each single mutant, suggesting that the ∆*sfgA* and ∆*vosA* mutations have synthetic negative effects on conidiogenesis.

### NsdD and VosA cooperatively repress *brlA* expression and conidiation

**Figure 2:**
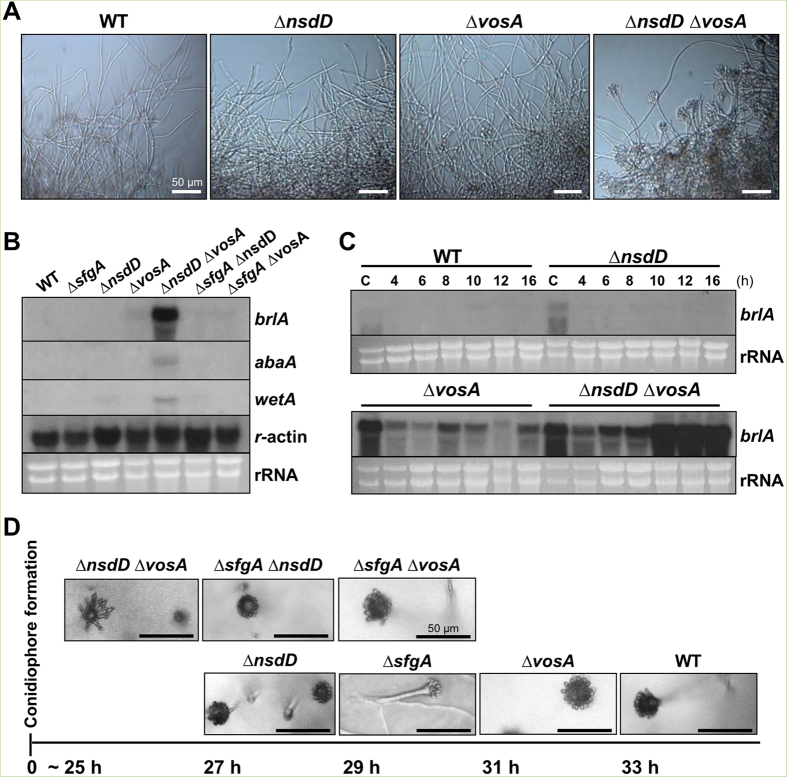
Accelerated conidiation by the lack of *vosA* and *nsdD*. (**A**) Photographs of WT (FGSC4), Δ*nsdD* (TNJ108), Δ*vosA* (THS15) and Δ*nsdD* Δ*vosA* (TMK11) hyphae at 16 h in liquid MMG. (Bar = 50 μm). (**B**) Levels of *brlA*, *abaA,* and *wetA* mRNA in designated strain grown in liquid submerged culture for 16 h. The γ-actin gene was used as a control. (**C**) Levels of *brlA* mRNA in WT, Δ*nsd*, Δ*vosA* and Δ*nsdD* Δ*vosA* strains in liquid submerged culture up to 16 h. C = conidia. (**D**) Time needed for the formation of the first conidiophore in single colonies of WT (FGSC4), Δ*nsdD* (TNJ108), Δ*vosA* (THS15), Δ*sfgA* (TNJ57), Δ*sfgA* Δ*nsdD* (TMK5), Δ*sfgA* Δ*vosA* (TMK10), and Δ*nsdD* Δ*vosA* (TMK11) on solid MMG. Photographs were taken at indicated time when the first conidiophore was visible. Numbers indicate the incubation time (h) after streak on solid MMG.

One approach to investigate elevated or hyper activation of conidiation is to grow the strains in liquid shake culture and check conidiophore development and mRNA levels of *brlA*. Under this condition, WT hardly ever produces asexual developmental structure. When WT, ∆*nsdD*, ∆*vosA,* ∆*sfgA*, ∆*sfgA* ∆*vosA,* ∆*sfgA* ∆*nsdD* and ∆*nsdD* ∆*vosA* strains were examined at 16 h liquid shake culture, only the ∆*nsdD* ∆*vosA* double mutant formed a high number of conidiophores (Fig. 2A). We then examined the mRNA levels of *brlA*, *abaA* and *wetA* in WT and various mutant strains at 16 h of vegetative growth, and found that only the Δ*nsdD* Δ*vosA* double mutant showed a high level accumulation of *brlA* mRNA (Fig. 2B). Accumulation of *abaA* and *wetA* mRNA was consistent with the *brlA* mRNA expression pattern in the Δ*nsdD* Δ*vosA* mutant. These led us to determine the levels of *brlA* mRNA in WT, Δ*nsdD*, Δ*vosA*, and Δ*nsdD* Δ*vosA* strains in conidia and very early phases of growth (4~16 h of liquid culture). As shown in Fig. 2C, the Δ*vosA* mutant displayed a high level of *brlA* mRNA in conidia and somewhat reduced levels of *brlA* mRNA in vegetative cells, lacking further activation of *brlA* expression. On the contrary, the Δ*nsdD* Δ*vosA* mutant exhibited a high level of *brlA* mRNA in conidia, and began to show induced activation of *brlA* expression even at 6 h of liquid culture, and a sudden strong activation of *brlA* expression at 10 h and thereafter. In fact, the Δ*nsdD* Δ*vosA* mutant formed conidiophores as early as 12 h of liquid culture (data now shown). These findings indicate that NsdD and VosA are major negative regulators of *brlA* expression and conidiation, and that the removal of the repressive effects imposed by NsdD and VosA might be a key factor determining the developmental competence.

We also check timing of conidiation on solid air-exposed culture condition. Somewhat consistent with the above findings, the time required for the first conidiophore formation in a colony derived from a single conidium on solid medium was about 25 h in the Δ*nsdD* Δ*vosA* mutant (Fig. 2D). The Δ*sfgA* Δ*nsdD* and Δ*nsdD* mutants showed initial conidiophore development at 27 h. The Δ*sfgA* Δ*vosA* and Δ*sfgA* mutants formed the first conidiophore at 29 h. The Δ*vosA* showed elaboration of conidiophore at ~31 h, whereas WT formed the first conidiophore at 33 h. These results suggest collectively that negative regulation of *brlA* by both NsdD and VosA is a key attribute determining the developmental competence.

### NsdD represses conidiation in *A. flavus* and *A. fumigatus*

**Figure 3:**
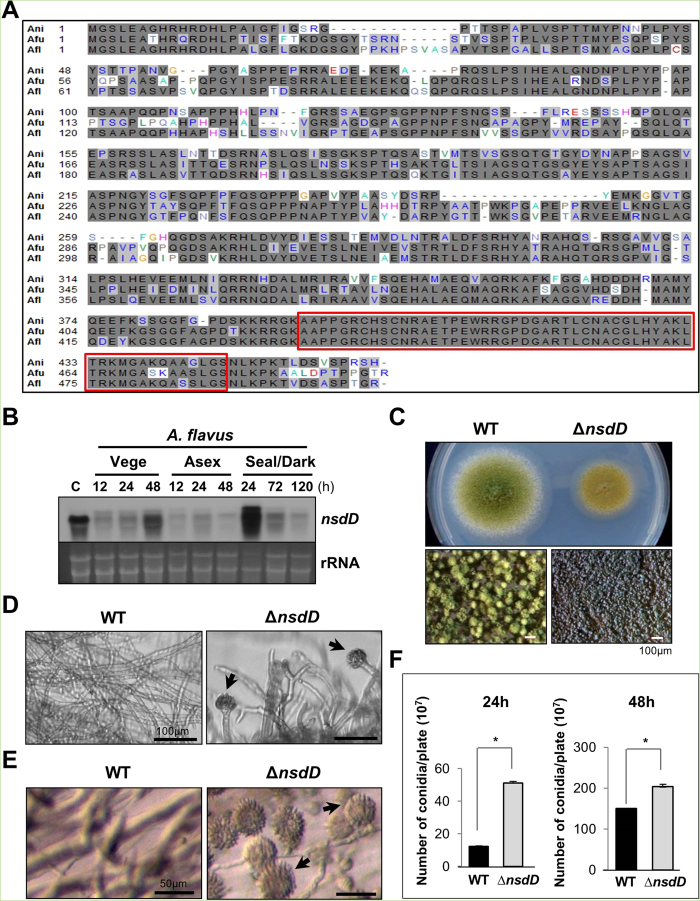
Characterization of *nsdD* in *A. flavus*. (**A**) Alignment the NsdD proteins of *A. fumigatus* (Afu3g13870), *A. flavus* (AFL2T_03635), and *A. nidulans* (AN3152). The red box indicates the highly conserved region. (**B**) Levels of *nsdD* mRNA during the lifecycle of *A. flavus* WT (NRRL3357). C = Conidia. The time (h) of incubation in liquid submerged culture (Vege), post asexual induction (Asex), and sealed/dark condition (Seal/Dark) is shown. Equal loading of total RNA was confirmed by ethidium bromide staining of rRNA. (**C**) Phenotypes of *A. flavus* WT and Δ*nsdD* (LNJ11) strains point inoculated on solid MMG with 0.1% yeast extract (YE) and incubated at 30 °C for 3 days. Close-up views (lower panel) of the center of individual colonies. (Bar = 100 μm). (**D**) Cells of *A. flavus* WT and Δ*nsdD* strains in liquid submerged culture grown for 28 h. Note the abundant formation of conidiophores in Δ*nsdD* strain (Bar = 100 μm). Conidiophore is marked by arrowhead. (**E**) Agar-embedded cells of *A. flavus* WT and Δ*nsdD* strains grown on solid MMG for 28 h. Abundant formation of conidiophores in Δ*nsdD* strain is evident. (Bar = 50 μm). (F) Quantitative analyses of conidiation in *A. flavus* WT and Δ*nsdD* strains. About 10^5^ conidia were spread on solid MMG with 0.1% YE, incubated for 24 and 48 h, and the conidia numbers per plate were counted in triplicates (*P < 0.001).

All *Aspergillus* species appear to have an ortholog of NsdD (AspGD; http://www.aspgd.org/). The predicted NsdD polypeptide, especially the GATA domain in the C-terminus, is highly conserved in *A. flavus* and *A. fumigatus* (Fig. 3A). We hypothesized that NsdD might play a similar repressive role in conidiation in these aspergilli.

In *A. flavus*, *nsdD* mRNA levels are high in conidia, and undulate during the lifecycle (Fig. 3B). The deletion of *nsdD* in *A. flavus* by replacing its coding region with the *pyrG*^+^ marker from *A. fumigatus*, caused restricted colony growth coupled with abnormal conidiophores compared to WT (NRRL3357; Fig. 3C). The size of the ∆*nsdD* conidiophores was averaged 45.81 μm, whereas WT conidiophore size was averaged 125.6 μm (P < 0.005; data not shown). This is consistent with the previous report demonstrating that NsdD is a major determinant of developmental morphogenesis^26^. We then examined levels of conidiation in varying ways, and found that the absence of *nsdD* resulted in hyper-active conidiation evidenced by the elaboration of a high number of conidiophores at 28 h of liquid shake culture (Fig. 3D), the formation of abundant conidiophores imbedded in agar at 28 h of solid culture (Fig. 3E), as well as enhanced production of conidia per plate (Fig. 3F). These results indicate that NsdD is a key repressor of conidiation in *A. flavus*.

**Figure 4:**
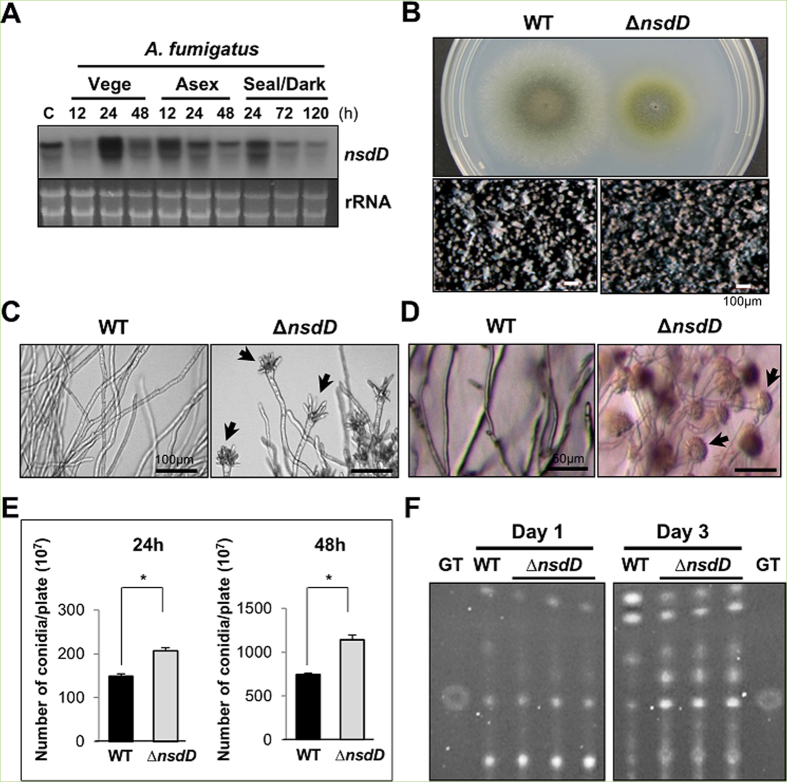
Characterization of *nsdD* in *A. fumigatus*. (**A**) Levels of *nsdD* mRNA during the life cycle of *A. fumigatus* WT (AFU293). C = conidia. See Fig. 3B legend for the times. Equal loading of total RNA was confirmed by ethidium bromide staining of rRNA. (**B**) Phenotypes of *A. fumigatus* WT and Δ*nsdD* (FNJ19) strain point inoculated on solid MMG with 0.1% YE and incubated at 37 °C for 3 days. Close-up views (lower panel) of the center of the colonies are shown (Bar = 100 μm). (**C**) Cells of *A. fumigatus* WT and Δ*nsdD* strains grown in liquid submerged culture for 19 h (Bar = 100 μm). Note abundant formation of aberrant conidiophores in the mutant. Conidiophore is marked by arrowhead. (**D**) Agar-embedded cells of *A. fumigatus* WT and Δ*nsdD* strains grown on solid MMG for 27 h (Bar = 50 μm). A high number of conidiophores was evident. (**E**) Quantitative analysis of conidiation: 10^5^ spores of WT and Δ*nsdD* strains were spread on solid MMG with 0.1% YE, incubated for 24 and 48 h, and the conidia numbers per plate were counted in triplicates (*P < 0.001). (**F**) Thin-layer chromatogram of CHCl_3_ extracts of *A. fumigatus* WT and Δ*nsdD* strains grown in liquid MMG with 0.5% YE for 1 and 3 days (stationary culture). Gliotoxin standard (GT) is shown.

In *A. fumigatus*, *nsdD* is somewhat constitutively expressed, and its mRNA levels are high in conidia (Fig. 4A). Similar to *A. nidulans* and *A. flavus*, the deletion of *nsdD* in *A. fumigatus* caused restricted hyphal growth (Fig. 4B), and early and uncontrolled activation of conidiation leading to the formation of conidiophores at 19 h liquid shake culture (Fig. 4C), and elaboration of a high number of conidiophores imbedded in agar at 28 h of solid culture (Fig. 4D), and enhanced production of conidia per plate (Fig. 4E). As the mycotoxin gliotoxin (GT) biosynthesis is activated by BrlA^25^,^27^, the deletion of *nsdD* resulted in elevated production of GT (Fig. 4F). These results indicate that NsdD functions as a negative regulator of conidiation and GT production in the opportunistic human pathogen *A. fumigatus*.

### NsdD directly binds to the *brlAβ* promoter region

**Figure 5:**
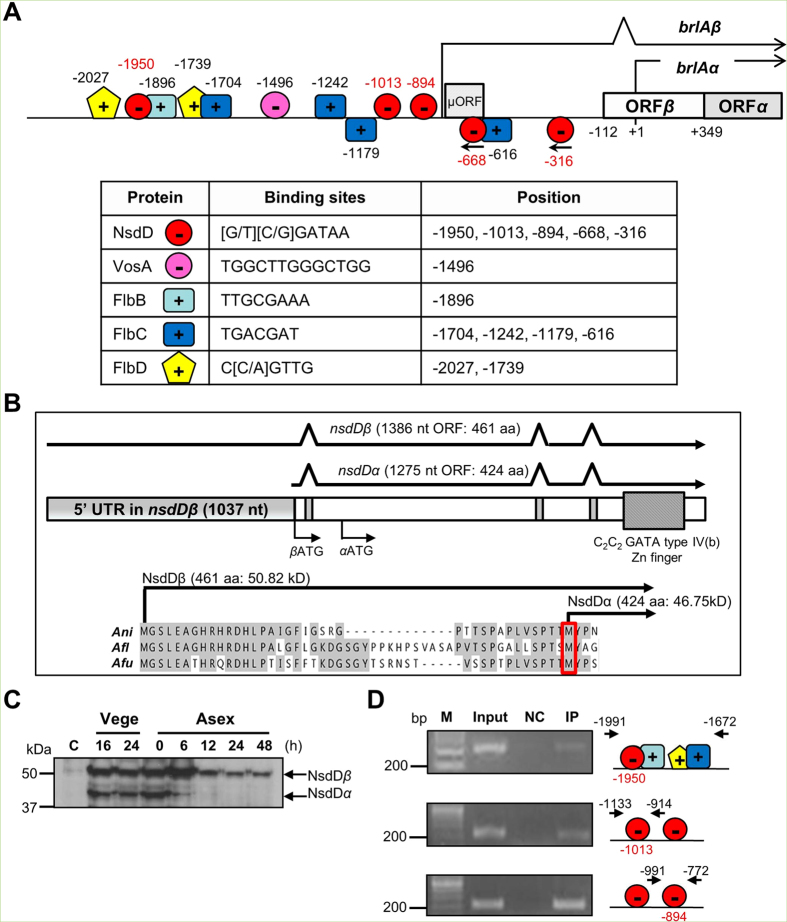
Regulatory elements of *brlA* and interaction of NsdD with the *brlA* upstream region. (**A**) A schematic diagram showing the binding regions (sequences) of VosA, FlbB, FlbC, and FlbD, and the putative GATA regions for NsdD. The *brlAα* transcription start site is denoted as “+1”. Hence, the BrlAβ and BrlAα translational start ATGs are at “−112” and “+349”, respectively. (**B**) Summary of the *nsdD* locus encoding two polypeptides. Gene structure was verified by sequence analyses of various cDNAs of *nsdD*. Start codon is assigned as ‘*β*ATG’ and ‘*α*ATG’. The predicted NsdDα polypeptide lacks the first 37 aa present in NsdDβ. (**C**) Western blot analysis of NsdDβ (~51 kDa) and NsdDα (~46 kDa) using anti-FLAG antibody and the TMK 13 strain. C = Conidia. Numbers indicate the time (h) of incubation in liquid submerged culture (Vege) and post asexual developmental induction (Asex). (**D**) Verification of NsdD binding to the *brlA* promoter by ChIP-PCR. The NsdD-ChIP was performed with 2 day-old conidia of TMK13. The PCR amplicons were separated on a 2% agarose gel. The chromatin sample before immuno-precipitation (IP) was used as a positive control (Input). The chromatin sample being incubated with beads alone without anti-FLAG antibody was used as a negative control (NC). Representative results and positions of each primer pair for PCR amplification are shown.

We previously reported that, in the FluG-mediated conidiation pathway, NsdD functions downstream of FlbE/B/D/C and upstream of *brlA*^21^. In a simplistic interpretation, we hypothesized that NsdD directly binds to upstream of the *brlA* coding sequence. To test this hypothesis, we first scanned the *brlA* promoter region spanning 2 kb with JASPAR CORE database (http://jaspar.genereg.net) to search potential GATA-TF binding sites. NsdD contains a zinc finger GATA binding domain at 394^th^–446^th^ amino acid at its C-terminus, which may interact with a core [A/T]GATA[A/G] consensus sequence. As shown in Fig. 5A, we identified five core GATA sequences located at the −1950 (+), −1013 (+), −894 (+), −668 (−), and −316 (−) nucleotide, where the transcription initiation site for *brlAα* is designated as +1 5. Binding sites of VosA, FlbB, FlbC, and FlbD in the *brlAβ* promoter are also marked in Fig. 5A 16,17,23.

Our previous study revealed that *nsdD* encodes two distinct transcripts designated as *nsdDβ* and *nsdDα*^21^. The *nsdDα* transcript specifically accumulates in conidia, whereas the *nsdDβ* constitutively accumulates throughout the lifecycle. Specific expression of *nsdDα* in conidia requires activity of both VosA and VelB during the formation of spores^23^. The *nsdDβ* and *nsdDα* transcripts contain 1,037 nt and 150 nt of 5′ untranslated region (UTR), respectively (Fig. 5B). Further analyses of cDNAs by RT-PCR indicate that these transcripts are predicted to encode the NsdDβ (461aa) and NsdDα (424aa) polypeptides, where NsdDα lacks the first 37 aa found in NsdDβ. To check the presence and expression levels of the predicted two NsdD proteins during the lifecycle, we carried out Western blot analysis employing a strain ectopically expressing NsdD::3XFLAG in Δ*nsdD* (TMK13, Table 1) and anti-FLAG antibody. We found that levels of both the NsdDβ and NsdDα proteins were very low in conidia, high in vegetative cells, then NsdDα became undetectable at 6 h post developmental induction (Fig. 5C). Employing the TMK13 strain and anti-FLAG antibody, we pulled-down the NsdD interacting DNAs in conidia, and PCR-amplified the five regions containing a core GATA site in the *brlA* promoter (ChIP-PCR). As shown in Fig. 5A,D, the three regions spanning −1,950, −1,013, and −894 containing the GATAA sequence in the + strand gave rise amplicons. However, the two regions containing GATAA in the − strand were not enriched by NsdD-ChIP. Taken together, while the precise NsdD binding sequence should be identified and validated, multiple NsdD might occupy the *brlAβ* promoter region. It can be further proposed that binding of NsdD and VosA to the *brlA* promoter results in the full repressive control of *brlA* expression, and the developmental competence might be determined by the removal of these key direct negative regulators of *brlA* (see Discussion).

### The positive roles of FlbC and FlbD in conidiation

**Figure 6:**
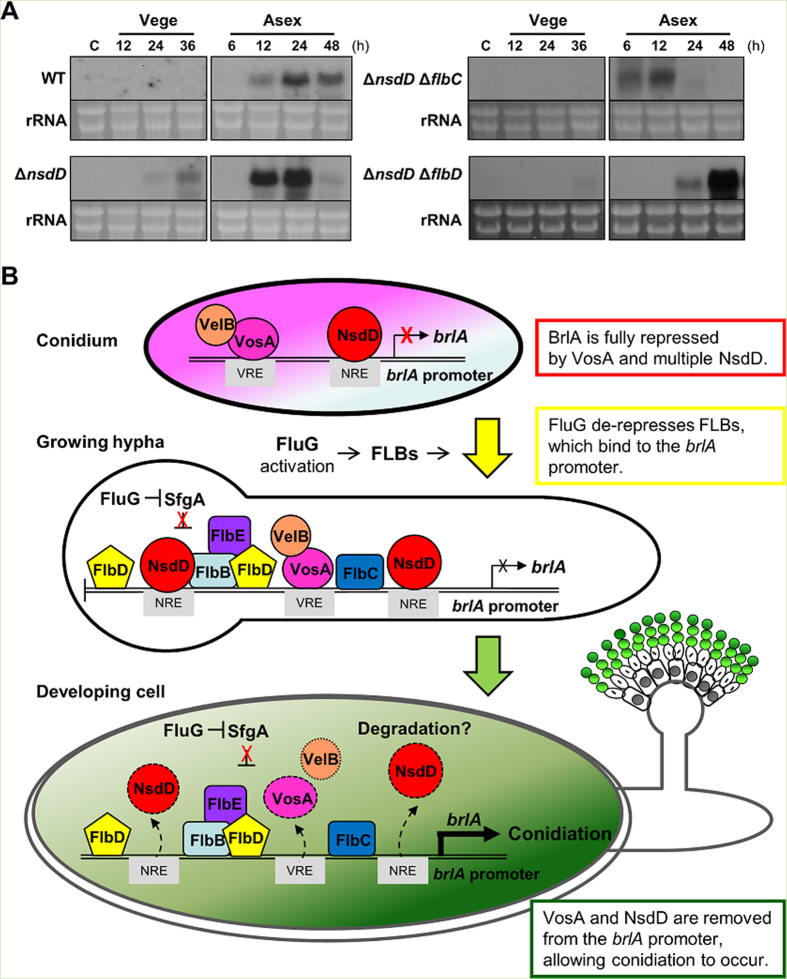
The role FlbC and FlbD in activating *brlA*, and a model for regulation of the commencement of conidiation. (**A**) Levels of *brlA* mRNA during the life cycle of WT (FSCG4), Δ*nsdD* (TNJ108), Δ*nsdD* Δ*flbC* (TNJ176) and Δ*nsdD* Δ*flbD* (TNJ178) strains are shown. The numbers indicate time (h) of incubation in liquid submerged culture (Vege), and post asexual developmental induction (Asex). C = conidia. Equal loading of total RNA was confirmed by ethidium bromide staining of rRNA. (**B**) A model depicting the roles of Flbs, VosA, and NsdD in governing the acquisition of developmental competence and the commencement of conidiation (see text).

We previously showed that the deletion of *nsdD* suppressed the conidiation defects caused by the absence of FLBs^21^. We further asked whether the primary role of FLBs is to remove the NsdD-mediated negative regulation or they play distinct roles in activating conidiation. This was done by determining timing and levels of *brlA* in the Δ*nsdD* single and ∆*flbC* ∆*nsdD* and ∆*flbD* ∆*nsdD* double mutants. As shown in Fig. 6A, *brlA* accumulation was not observed in WT vegetative conditions, and was slightly increased 12 h post asexual developmental induction. The deletion of *nsdD* caused *brlA* mRNA accumulation at 36 h in submerged culture (vegetative), and at high levels at 12 and 24 h post induction. Importantly, we found that, even in the absence of *nsdD*, the deletion of *flbC*, or *flbD* resulted in significantly reduced and delayed accumulation of *brlA* mRNA at 24 and 48 h post induction. These results indicate that these positive regulators play distinct positive roles in activating *brlA* expression, and their activities are needed for full activation of *brlA*.

## Discussion

Conidiation in *Aspergillus* occurs as an integral part of the life cycle primarily controlled by the intrinsic genetic program rather than as a response to unfavorable environmental conditions^1^. Neither the concentration of a limiting nutrient such as glucose or nitrogen source, nor continuous transfer to new medium modifies the timing with which cells become competent to develop^1^,^28^.

Given that the timing of competence acquisition is endogenous and genetically determined, one can ask how the fungus keeps track of the time that has transpired following germination. Timberlake^29^ came up with an explanation for this by proposing a repressor of conidiation, which becomes diluted during early growth. In this model, a fixed amount of repressor would be produced during the final stages of conidium differentiation and stored in the spore. During the (~18 h of) vegetative growth, such a repressor is diluted to a critical concentration before development can proceed. Timberlake further speculated that it could be a negatively acting TF that prevents expression of genes required for conidiation, e.g., *brlA*, and mutational inactivation of such a repressor would be expected to lead to precocious development.

Indeed, collectively, our studies have revealed that there are at least three negative regulators of conidiation, and that a key event for the acquisition of developmental competence is to remove the repressive effects imposed by NsdD and VosA. We further have found that the positive upstream regulators are needed for maximum level conidiation, but not for the commencement of development. This is based on the fact that the deletion of *nsdD* could bypass the need for *fluG*, *flbB*, *flbE*, *flbD*, and *flbC*, but not *brlA*, in conidiation^21^. Importantly, for the first time, we demonstrated that NsdD physically binds to three different regions in the *brlAβ* promoter, further supporting the idea that NsdD directly (rather than indirectly) represses the onset of *brlAβ* expression and conidiation.

NsdD is a GATA TF with a highly conserved DNA-binding domain consisting a Cys2-Cys2 type IV zinc finger in its C-terminal basic region^24^. GATA TFs (GATA-1 ~ GATA-6) bind to a DNA sequence called a GATA motif [(A/T)GATA(A/G)] present in the regulatory regions of their target genes through two zinc finger domains^30^. Our *in vivo* NsdD-DNA interaction analyses in conidia suggest that the NsdD responsive elements (NREs) might be composed of the GATAA core sequence. As shown in Fig. 5, the three regions containing GATAA in the + strand, but not the two regions with GATAA in the − strand, can be enriched by NsdD-ChIP. The GATAA sequence at −1950 is positioned between the predicted binding sites for the two key UDAs, FlbB and FlbD^13^,^17^. The two sites at −1013 and −894 may overlap with the RNA polymerase binding region (the *brlAβ* transcript is marked by the arrow-head line in Fig. 5A). A revised VosA responsive element (VRE; TGGCTTGGGCTGG) is positioned at −1,496 between the two predicted FlbC binding sites^16^,^23^. Thus, binding of multiple NsdD and one VosA-VelB in the promoter region of *brlAβ* may effectively inhibit the initiation of *brlAβ* transcription. In fact, as shown in Fig. 2A–D, the absence of both *nsdD* and *vosA* resulted in near constitutive expression of *brlA* throughout the life cycle, and abundant/precocious conidiophore development in liquid culture (as early as 12 h). However, the observation that about 25 h is required for the elaboration of the first conidiophore in the Δ*nsdD* Δ*vosA* mutant colony derived from a conidium (Fig. 2D) suggests that a single spore must undergo vegetative growth for a certain period even in the absence of the key negative regulators of conidiation.

The NsdD polypeptide(s) is highly conserved in most (if not all) *Aspergillus* species (Fig. 3A) and other fungi including *Penicillium*, *Coccidioides*, *Ajellomyces*, and *Fusarium* (not shown). Moreover, the *A. fumigatus* and *A. flavus nsdD* genes appear to encode two transcripts (and polypeptides; see the second Met position Fig. 3A,B and 4A). We demonstrated that the role of NsdD in negatively controlling *brlA* and conidiation is conserved in these two species. In *A. fumigatus*, there are four GATAA sequences at −2276, −1148, −976, and −932, where the BrlA ATG is +1. In *A. flavus*, two GATAA sequences are present at −1413 and −1389, where the BrlA ATG is +1. In both cases, the deletion of *nsdD* resulted in precocious and enhanced conidiation, which is consistent with a previous report^31^. Early and increased production of GT in the *A. fumigatus nsdD* mutant can be explained by precocious and enhanced expression of BrlA, which in turn directly activates gliotoxin biosynthesis^27^,^32^.

Tight repression of *brlA* in a conidium and for a certain period of vegetative growth is important for the fitness of *Aspergillus* fungi. Adams and Timberlake^33^ showed that overexpression of *brlA* in vegetative cells resulted in complete cessation of growth and generalized losses of protein and RNA. Collectively, we present a genetic model depicting the negative and positive regulations and the commencement of conidiation in *A. nidulans* (Fig. 6). During the formation of conidia, VosA and VelB are activated by AbaA^34^, which in turn activate expression of the lower transcript of *nsdD* in conidia^23^. VosA and multiple NsdD are bound to the upstream regulatory region of *brlA*, which confers full repression of *brlA* and conidiation. SfgA acts as an upstream negative regulator of conidiation functioning downstream of FluG^20^. During early phase of vegetative growth, FluG accumulates to a certain level, which then removes the repressive effects of SfgA, thereby allowing UDAs (FlbB/D and FlbC) to function. Acquisition of the developmental competence might also involve the translocation of FlbB to the hyphal tip, became transcriptionally competent, then entering into the nucleus^35^. In order for activated FlbB-FlbD^17^ and FlbC to trigger *brlA* expression and conidiation, both NsdD and VosA need to be removed from the *brlA* promoter. Currently, we do not know how NsdD and VosA are displaced from the *brlA* promoter. One possible explanation is degradation of the VosA and NsdD proteins. Upon removal of NsdD and VosA coupled with the cooperative activity of FLBs, *brlAβ* is expressed above threshold, which then fully activates itself and *brlAα*, triggering development of conidiophores^21^. While not shown in the model, activated BrlA leads to expression of AbaA, which in turn activates expression of VosA, VelB, and WetA in phialides and conidia (see Fig. 1B). The VosA-VelB heterodimer shuts off expression of *brlA* and *β*-glucan biosynthetic genes, and activates genes associated with trehalose biogenesis and *nsdDα* in conidia, allowing full repression of *brlA* for next generation^19^,^34^,^36^.

Finally, while we presented a simplified single-path model for conidiation, it is important to note that that regulation of development is a complex multi-degree process involving both activation of the FluG-initiated conidiation pathway and inhibition of FadA-mediated G protein signaling pathway for vegetative growth^37^,^38^,^39^,^40^,^41^. In *A. nidulans*, various G protein mutants displayed precocious activation of conidiation^42^,^43^,^44^. Moreover, high level accumulation of *brlA* alone might not be sufficient to trigger conidiation as shown in our *ricA* study^45^. In *A. fumigatus*, various developmental regulators including *velvet* proteins, G-proteins, and RAS proteins govern conidiation (reviewed in ref. 46 and 47). Additional studies integrating genome-wide and systems analyses are in progress to better address the developmental control mechanisms in *Aspergillus*.

## Methods

### Fungal strains and culture conditions

The *Aspergillus* strains used in this study are listed in Table 1. *A. nidulans* strains were grown on solid or in liquid minimal medium with 1% glucose (MMG) with supplements as described previously^48^ at 7 °C. To determine the numbers of conidia in WT and mutant strains, approximately 10^5^ spores were spread onto solid MMG and incubated at 37 °C for 2 days. The conidia were collected from the entire plate and counted using a hemocytometer. To check elaboration of conidiophores in liquid submerged culture, conidia (10^6^/ml) of individual strains were inoculated in liquid MMG and incubated at 37 °C, 220 rpm. For Northern blot analyses, samples were collected as described^49^. Briefly, for vegetative growth, conidia (10^6^/ml) of strains were inoculated in liquid MMG and cultured at 37 °C, 220 rpm. Samples of liquid submerged culture were collected at designated time points. Induction of asexual development or sexual development was done as described previously^49^.

*A. flavus* and *A. fumigatus* strains were grown on solid or in liquid MMG with 0.1% yeast extract (YE, v/v) and supplements as described^48^,^50^, at 30 °C and 37 °C, respectively. To check elaboration of conidiophores in liquid submerged culture, conidia (2 × 10^5^/ml) of individual strains were inoculated in liquid MMG with 0.5% YE and incubated, 220 rpm. For developmental induction, vegetative cells were collected and transferred to solid medium, and the culture plates were air exposed for asexual developmental induction or tightly sealed induction in dark condition as described^49^.

### Construction of *A. nidulans* strains

The oligonucleotides used in this study are listed in Table S1. The double joint PCR (DJ-PCR) method^51^ was used to generate the ∆*sfgA* ∆*vosA*, ∆*nsdD* ∆*vosA* and ∆*sfgA* ∆*nsdD* mutants. Both 5′ and 3′ flanking regions of the *sfgA* and *nsdD* genes were amplified from genomic DNA of FGSC4 using OMK556;OMK557 and OM558;OMK559 (for *sfgA*), and OMK562;OMK563 and OMK564;OMK565 (for *nsdD*). The *A. nidulans pyroA*^+^ marker was amplified with the primer pair ONK395;ONK396. The final DJ-PCR *sfgA* deletion construct was amplified with OMK560;OMK561, and the *nsdD* deletion construct was amplified with OMK566;OMK567. The *sfgA* deletion amplicon was introduced into THS15.1 to generate the ∆*sfgA* ∆*vosA* mutant. The *nsdD* deletion amplicon was introduced into THS15.1 and TNJ57 to generate the ∆*nsdD* ∆*vosA* and ∆*sfgA* ∆*nsdD* mutants, respectively. Protoplasts were generated using the Vinoflow FCE lysing enzyme (Novozymes)^52^. At least three independent deletion mutant strains were isolated. To complement ∆*nsdD* and epitope-tag NsdD, the FGSC4 *nsdD* fragment including its 2kb 5′ and coding regions was amplified with the primer pair OMK574;OMK575, digested with *Pst*I and *Not*I, and cloned into the pHS13 vector^34^, which contains ^3/4^*pyroA*^53^, a 3xFLAG tag, and the *trpC* terminator. The resulting plasmid pMK20 was then introduced into the recipient ∆*nsdD* strain TNJ108, and several TMK13 class transformants expressing the WT NsdD fused with the 3XFLAG tag under its native promoter have been isolated and confirmed.

### Construction of *A. flavus* and *A. fumigatus* strains

The *nsdD* gene was deleted in *A. flavus* NRRL3357.5 (*pyrG*^−^)^54^ and *A. fumigatus* AFU293.1 (*pyrG*^−^)^55^ employing DJ-PCR^51^. In *A. flavus* strain, the 5′ and 3′ flanking regions of the *nsdD* gene were amplified using A. *flavus* WT (NRRL3357) genomic DNA with the primer pairs ONK1037;ONK1038 and ONK1039;ONK1040. The *A. fumigatus pyrG*^+^ marker was amplified from *A. fumigatus* WT (AFU293) genomic DNA with the primer pair OMK589;OMK590. The 5′ and 3′ flanking regions of *nsdD* were fused to the marker, and the resulting fusion product was further amplified by the nested primer pair ONK1041;ONK1042. The final deletion construct was introduced into *A. flavus* NRRL3357.5, and the ∆*nsdD* mutant (LNJ11) was isolated and confirmed by PCR followed by restriction enzyme digestion^51^.

In *A. fumigatus* strain, the 5′ and 3′ flanking regions of *nsdD* were amplified from A. *fumigatus* WT (AFU293) with the primer pairs ONK1043;ONK1044 and ONK1045;ONK1046. The *A. nidulans pyrG*^+^ marker was amplified from FGSC4 genomic DNA with the primer pair OHS696;OHS697. The 5′ and 3′ flanking regions of *nsdD* were fused to the marker, and the fusion product was further amplified by the nested primer pair ONK1047;ONK1048. The final deletion construct was introduced into *A. fumigatus* AFU293.1, and the ∆*nsdD* mutant (LNJ12) was isolated and confirmed by PCR followed by restriction enzyme digestion^51^. At least three independent deletion strains were isolated and confirmed.

### Nucleic acid isolation and manipulation

Genomic DNA and total RNA isolation was carried out as previously described^18^,^56^. In Northern blot analyses, DNA probes were prepared by PCR amplification of the coding region of individual genes with appropriate oligonucleotide pairs using FGSC4 genomic DNA as template (Table S1). Probes were labelled with ^32^P-dCTP (PerkinElmer) using Random Primer DNA Labeling Kit (Takara) and purified by illustra MicroSpin G-25 columns (GE Healthcare).

### Protein extraction and Western blot analysis

Western blot analysis of NsdD was performed using conidia, vegetative cells, and developing cells of TMK13. Individual samples including 2 d old conidia (2 × 10^8^) were collected and resuspended in the lysis buffer (50 mM Tris-HCl [pH7.2], 150 mM NaCl, 1% Triton X-100, 1 mM EDTA), and homogenized by using a Mini Bead beater and 0.2 ml of silica-zirconium beads (Biospec)^57^. Protein concentration was colorimetrically determined using a Protein Assay System (BioRad). Approximately 10 μg of total proteins per a lane were separated on 10% SDS-PAGE gel and transferred onto immobilon-P membrane (Millipore). The membrane was incubated with the mouse monoclonal Anti-FLAG antibody (M2 clone, Sigma-Aldrich), and then subsequently incubated with a secondary antibody conjugated with horseradish peroxidase, HRP-Goat anti-mouse IgG (Millipore). The membrane was developed using enhanced chemilluminescence reagents (Amersham Biosciences).

### Chromatin immunoprecipitation assay

Briefly, two days-old conidia (1 × 10^9^ conidia) of TMK13 were crosslinked with fresh 1% formaldehyde for 30 min at RT. Then, 125 mM of glycine buffer was added to stop the cross-linking reaction. The conidia were resuspended in spore lysis buffer (50 mM Tris-HCl [pH 7.5], 150 mM NaCl, 5 mM EDTA, 15 mM EGTA, 0.5% Triton X-100, 0.1% sodium deoxycholate, 0.1% SDS, 1x protease inhibitor cocktail). Resuspended samples were mixed with silica beads and broken by a mini-bead beater for 3 min. Subsequently, the samples were sonicated for five cycles (30 s on, 60 s off) with a sonifier microtip at 70% amplitude and level 5 of output control^57^. The lysates were finally diluted in ChIP dilution buffer (0.01% SDS, 1.1% Triton X-100, 1.2 mM EDTA, 16.7 mM Tris/HCl, 167 mM NaCl, pH 8.0), and then the lysates were applied for ChIP assays according to the manufacturer’ instructions with a slight modification (MAGnify Chromatin Immunoprecipitaion System, Invitogen). The lysate was reacted with 1 μg of the mouse monoclonal Anti-FLAG antibody (Sigma-Aldrich). As a negative control, the chromatin extract was incubated with 1 μg of Anti-rabbit IgG in this assay. Individual input DNA samples before immune-precipitation (IP) were used as positive controls. Finally, the enriched DNA was purified and used as a template for PCR reactions with the GO *Taq* DNA polymerase (Promega). The primer sets used for PCR are shown in Supplementary Table S1.

### Gliotoxin analysis

Gliotoxin (GT) production was analyzed by thin-layer chromatography (TLC) as described^55^. Briefly, spores (10^5^/ml) of *A. fumigatus* strains were inoculated into 2 ml liquid MMG with 0.5% YE and stationary cultured at 37 °C up to 3 days. GT was extracted by CHCl_3._ Each sample was loaded onto a TLC silica plate including a fluorescence indicator (Kiesel gel 60, 0.25 mm; Merck). As a positive control, ~1 μg of GT standard (Sigma-Aldrich, St. Louis, MO) was applied. The plate was then developed with toluene-ethyl acetate-formic acid (5:4:1, v/v/v) as mobile phase, where the R(*f* ) value of GT was ~0.61. Photographs of TLC plates were taken following exposure to UV (365 nm) using a Sony DSC-T70 digital camera. This analysis was performed in triplicates.

### Microscopy

The colony photographs were taken by using a Sony digital camera (DSC-F28). Photomicrographs were taken using a Zeiss M^2^ Bio microscope equipped with AxioCam and AxioVision (Rel. 4.8) digital imaging software.

## Additional Information

**How to cite this article**: Lee, M.-K. *et al*. Negative regulation and developmental competence in *Aspergillus*. *Sci. Rep.* **6**, 28874; doi: 10.1038/srep28874 (2016).

## Supplementary Material

Supplementary Table S1

## Figures and Tables

**Table 1:** *Aspergillus* strains used in this study.

Strain name	Relevant genotype	References
FGSC4	*A. nidulans* wild type	FGSC^b^
RJMP1.59	*pyrG89*; *pyroA4*	58
TNJ36	*pyrG89; AfupyrG*^+^; *pyroA4*	22
THS15	*pyrG89*; *pyroA4*; Δ*vosA*::*AfupyrG*^+^	34
TNJ57	*pyrG89*; Δ*sfgA*::*AfupyrG*^+^; *pyroA4*	45
TNJ108	*pyrG89*; *pyroA4*; Δ*nsdD*::*AfupyrG*^+^	21
TNJ176	*pyrG89*; *pyroA4*; Δ*nsdD*::*pyroA*^+^; Δ*flbC*::*AfupyrG*^+^	21
TNJ178	*pyrG89*; *pyroA4*; Δ*nsdD*::*pyroA*^+^; Δ*flbD*::*AfupyrG*^+^	21
TMK5	*pyrG89*; Δ*sfgA*::*AfupyrG*^+^; *pyroA4*; Δ*nsdD*::*pyroA*^+^	This study
TMK10	*pyrG89*; Δ*sfgA*::*pyroA*^+^; *pyroA4*; Δ*vosA*::*AfupyrG*^+^	This study
TMK11	*pyrG89*; *pyroA4*; Δ*nsdD*::*pyroA*^+^; Δ*vosA*::*AfupyrG*^+^	This study
TMK13	*pyrG89*; 3/4*pyroA4*::*nsdD*(p)::*nsdD*::FLAG_3X_::*trpC*(t)::*pyroA*^+^^c^; Δ*nsdD*::*AfupyrG*^+^	This study
NRRL3357	*A. flavus* wild type	FGSC^b^
NRRL3357.5	*AflpyrG*^−^	54
LNJ11	*AflpyrG*^−^; Δ*nsdD*::*AfupyrG*^+^	This study
AFU293	*A. fumigatus* wild type	59
AFU293.1	*AfupyrG1*^−^	55
FNJ19	*AfupyrG1*^−^; Δ*nsdD*::*AnipyrG*^+^	This study

^a^All *A. nidulans* strains carry the *veA*^+^ allele.

^b^Fungal Genetic Stock Center.

^c^The 3/4 *pyroA* marker restores *pyroA*^+^ when it integrates into the *pyroA4* locus by a single cross-over.
