# Supplementary material for: Negative regulation and developmental competence in Aspergillus
Source: Sci Rep. 2016 Jul 1;6:28874. doi: 10.1038/srep28874 (PMC4929475; doi:10.1038/srep28874)
Supplement: Supplementary Table S1 [file srep28874-s1.pdf]

Negative regulation and developmental competence in *Aspergillus*

Mi-Kyung Lee[1,2], Nak-Jung Kwon[1, †], Im-Soon Lee[2], Seunho Jung[3], Sun-Chang Kim[4], and Jae-Hyuk Yu[1, *]

[1]Department of Bacteriology, The University of Wisconsin-Madison, Madison, Wisconsin 53706, [2]Department of Biological Sciences and Center for Biotechnology Research, [3]Department of Bioscience and Biotechnology and Center for Biotechnology Research, Institute for Ubiquitous Information Technology and Applications (UBITA), Konkuk University, Seoul 143-701, Republic of Korea, [4]Department of Biological Sciences, Korea Advanced Institute of Science and Technology, Dae-Jeon, Republic of Korea

[*] **Corresponding Author:**
Dr. Jae-Hyuk Yu
Departments of Bacteriology and Genetics
1550 Linden Drive, Madison, 53706, USA
Tel: 608-262-4696
Fax: 608-262-9865
Email: jyu1@wisc.edu

†. Present address: Macrogen Co, Seoul, Korea.

Running title: Fungal Developmental Competence

Supplementary Table S1. Oligonucleotides used in this study

Name	Sequence (5' → 3')	Purpose
OMK556	AACGCAACGCAAAGGATAGCGAGC	5' flanking of *sfgA*
OMK557	*ACTTCTGCAGTCGGAATTGGCCTG*AGAGAAAGATCGACGGTGGGAGAG	3' *sfgA* with *pyroA* tail
OMK558	*TGGTGAGAACACATGCACAACTTG*TTGACCGGACCATACACCTCGCAC	5' *sfgA* with *pyroA* tail
OMK559	AGCAGACGTTCACTCAGAGCCACC	3' flanking of *sfgA*
OMK560	AATTTGGTAGTTGCGCCTACGGC	5' nest of *sfgA*
OMK561	GTCGCAGAGGCGTAGCTCTACAG	3' nest of *sfgA*
OMK562	AGTTCGCAGCCTGTAAAGCTTCTG	5' flanking of *nsdD*
OMK563	*ACTTCTGCAGTCGGAATTGGCCTGAG*TGATCCCATAGCGCAGGCAATC	3' *nsdD* with *pyroA* tail
OMK564	*TGGTGAGAACACATGCACAACTTG*ACCGAGGAGTCATTAACACGCTAG	5' *nsdD* with *pyroA* tail
OMK565	ACGGTATGGAGAGGAAACACGGATG	3' flanking of *nsdD*
OMK566	TAAGCTGATCGATCGTCCGCTCTC	5' nested of *nsdD*
OMK567	TCACTTGAGCTGGTTATGTGGTGG	3' nested of *nsdD*
ONK1037	TCCCTCGAGGCAGTTGTCTCCTAG	5' flanking of *AflnsdD*
ONK1038	*GCTTTGGCCTGTATCATGACTTCA*TCCGGTTATATCCTTGCGATGTCG	3' *AflnsdD* with *Afupyr*G tail
ONK1039	*ATCGACCGAACCTAGGTAGGGTA*AACGAAGAAAGGCTTTGGGATTAG	5' *AflnsdD* with *Afupyr*G tail
ONK1040	AGAGCCTCAATTCTTGGGAATGAC	3' flanking of *AflnsdD*
ONK1041	TGTGGTCTTCCTCTATCATCTATC	5' nest of *AflnsdD*
ONK1042	TCAGTATCTAGTTAGAACCACTGG	3' nest of *AflnsdD*
OMK574	AGAT**CTGCAG**TGCAGCAGTAGTAGAGTGTATCAG	5' *nsdD with Pst*I
OMK575	AGAT**GCGGCCGC**ATGACTCCTCGGTGACACCGAGTC	3' *nsdD* with *Not*I
OMK589	GCTGAAGTCATGATACAGGCCAAA	5' *AfupyrG* marker
OMK590	ATCGTCGGGAGGTATTGTCGTCAC	3' *AfupyrG* marker
ONK395	ATCTCATGGGTGCTGTGCGAAAGG	5' *AnipyroA* marker
ONK396	TTGCATCGCATAGCATTGCATTGC	3' *AnipyroA* marker
OMK578	CTGGCAGGTGAACAAGTC	5' *brlA* probe
OMK579	AGAAGTTAACACCGTAGA	3' *brlA* probe
ONK1043	AGTAACTCAGGTACTAGTCCGTAG	5' flanking of *AfunsdD*
ONK1044	*TTTGTAGGCTTTGGGCTGTTCACAA*TGTCACTTTGAGTATTCCAGTCTC	3' *AfunsdD* with *AnipyrG* tail
ONK1045	*CTGATCTACCCCTTGGAACGCAGCA* GTTTGCGGCGTTGAAAAGGCATT	5' *AfunsdD* with *AnipyrG* tail

ONK1046	TTGCAGCTGGAGAAGTACTAGACG	3' flanking of *AfunsdD*
ONK1047	TAGTCATAGCTAATCTCTCCCTCC	5' nest of *AfunsdD*
ONK1048	TTCTAGTGCGACATTGGAGTCAGG	3' nest of *AfunsdD*
OMK587	AGCTCTTCAGAATACGTC	5’ *abaA* probe
OMK588	GTTGTGAGATGCCTCCAT	3’ *abaA* probe
OMK580	CAGTACGTCAATATGGAC	5’ *wetA* probe
OMK581	GTGAAGTTGACAAACGAC	3’ *wetA* probe
OJA174	CATTGAGCACGGTGTTGT	5’ gamma-actin [60]
OJA175	ATCCCTTGATCTCGTTTG	3’ gamma-actin
OMK546	AACGCCTTCTCCAACCG	5’ NsdD_ChIP PCR (-1991)
OMK547	TTGCCTCATTCCTCTCC	3’ NsdD_ChIP PCR (-1672)
OMK556	GCGAGTCTTCTGATACCG	5’ NsdD_ChIP PCR (-1133)
OMK557	CTTTCTGCGGTGGGCCTC	3’ NsdD_ChIP PCR (-914)
OMK558	GTCGGGAGATGAAGAAAT	5’ NsdD_ChIP PCR (-991)
OMK559	AGCAGAGAGAAAGTGGCA	3’ NsdD_ChIP PCR (-772)
OMK436	CCCTTTTACCACTTTGGGC	5’ NsdD_ChIP PCR (-751)
OMK437	GATCAGATGAGGGCCAGCA	3’ NsdD_ChIP PCR (-512)
OMK438	GCGTTTAAGGGCGGGTCTA	5’ NsdD_ChIP PCR (-391)
OMK439	AGCAAAGGCTGAAGGAGG	3’ NsdD_ChIP PCR (-152)

[a]Tail sequence is in italic.

[b]Restriction enzyme site is in bold.
